# The Link between ENSO-like Forcing and Hydroclimate Variability of Coastal East Asia during the Last Millennium

**DOI:** 10.1038/s41598-017-08538-1

**Published:** 2017-08-15

**Authors:** Jungjae Park, Jiwoo Han, Qiuhong Jin, Junbeom Bahk, Sangheon Yi

**Affiliations:** 1 Department of Geography, Seoul National University, Sillim-dong, Gwanak-gu, Seoul, 151-742 Republic of Korea; 2 Institute for Korean Regional Studies, Seoul National University, Sillim-dong, Gwanak-gu, Seoul, 151-742 Republic of Korea; 3 Geo-Environmental Hazards & Quaternary Geology Research Center, Korea Institute of Geoscience and Mineral Resources, Daejeon, 305-350 Republic of Korea

## Abstract

Inconsistent reconstructions of East Asian hydroclimate for the last millennium significantly limit our understanding of the mechanisms behind climate variability during the medieval climate anomaly (MCA) and little ice age (LIA) in the region. In this study, we present new high-resolution multiproxy records (diatom, δ^13^C, C/N, TS) from the Mulyoungari swamp, Jeju Island, South Korea. Our results indicate that El Niño southern oscillation-like variations caused the dry MCA/wet LIA pattern in the study area. Recent paleo-ENSO studies generally support the hypothesis that the MCA was characterized by more persistent El Niño-like conditions. During El Niño events, the genesis of typhoons affecting coastal East Asia tends to diminish because of warm anomalies of eastern tropical Pacific (ETP) SSTs and downward motions over the western tropical Pacific. Therefore, coastal East Asia likely experienced a decline in typhoon-related precipitation during the MCA, in contrast to monsoon-dominated northern China. Our results additionally imply that SST anomalies in the ETP need to be carefully checked to better understand current hydroclimate variability in coastal East Asia, one of the most populated areas on earth.

## Introduction

Many paleoclimatologists have been particularly interested in the climate of the last millennium since information regarding past climate is invaluable for predicting future climate changes and its impact on societies^1–5^. Attempts have been made to reconstruct paleoenvironmental history for the medieval climate anomaly (MCA; ca. 900–1350 CE) to assess the possible outcomes of current global warming^6, 7^. The little ice age (LIA; ca. 1350–1850 CE) also has long been investigated because historical documents clearly indicate that its unfavorable climate conditions substantially undermined human societies across the Northern Hemisphere^8–10^.

A number of paleoclimatological studies have reported East Asian climate variation of the last millennium^11–23^. It is widely agreed that atmospheric temperatures were relatively low (high) over East Asia during the LIA (MCA)^14, 24^. However, inconsistent hydroclimate reconstructions in the region limit an understanding of the mechanisms behind LIA and MCA precipitation^25, 26^. For example, central Asia and northwestern China were likely wet during the LIA due to reduced solar output and consequent southward migration of westerlies^27^. Meanwhile, summer monsoon activity may have been weakened (strengthened) in northern (southern) China by more southerly position of the Inter Tropical Convergence Zone (ITCZ)^25, 26^. East Asia could thus be hydro-climatologically divided into a westerlies-dominated region and a monsoon-dominated region^25^. However, substantial areas remain unclearly defined by such a broad classification.

In particular, paleoclimate of coastal East Asia cannot be fully understood without exploring oceanic influence. Previous proxy and reviewing studies have so far not seriously examined oceanic forcing^25^ despite its significant influence on present East Asia climate^28^. Coastal cities with fast growing populations are highly susceptible to current warming-induced increases in extreme events such as river floods and storm surges^29^. Even though the hydroclimate history of the last millennium is needed to address such climate crisis issues, it has been rarely reconstructed in coastal East Asia. This is partly associated with the difficulty to obtain late Holocene sedimentary records with no indication of agricultural disturbance. Recently, El Niño southern oscillation (ENSO) variability was suggested to have principally driven mid- to late-Holocene climate shift in coastal East Asia^30, 31^. Therefore, hydroclimate conditions in the region during the MCA and LIA were also likely linked to tropical pacific SST variability.

In this study, we present high-resolution multiproxy records (diatom, C/N, TS, δ^13^C) of the last millennium climate from the Mulyoungari swamp, Jeju Island, South Korea. The ~4000 year pollen records of the same sediment core have already been reported^30, 31^. Here, new proxy records, pollen data, and historical evidence are discussed together to address their paleoclimatic implications for the last millennium. The aims of this study are (1) to reconstruct the climate variability of MCA and LIA in coastal East Asia using multiproxy sedimentary records, (2) to identify the influence of oceanic forcing, and (3) to examine the possible mechanisms behind it.

### Site descriptions and modern climate

**Figure 1:**
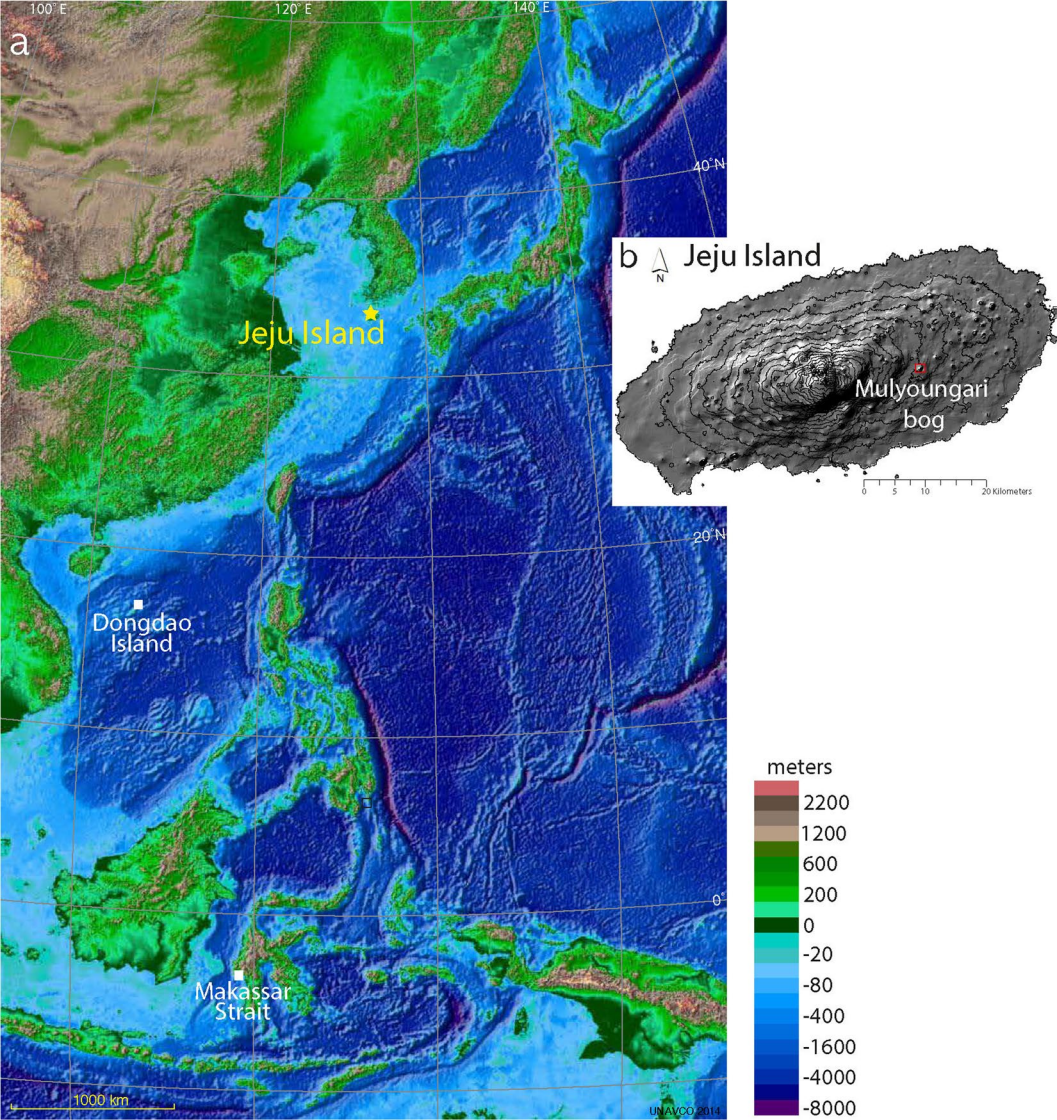
(**a**) Locations of the study site and paleoclimate records used in this study: Macassar Strait, Indonesia^60^ and Dongdao Island in South China Sea^59^ (see Fig. 4c,d). This map was modified from the UNAVCO Jules Verne Voyager (UNAVCO Inc., jules.unavco.org) based on Generic Mapping Tools (GMT-5; gmt.soest.hawaii.edu) (**b**) Location of the Mulyounari swamp in Jeju Island. The contour map was generated using software ArcGIS 10.1 (www.arcgis.com).

The Mulyoungari crater swamp (33°22′09′′N, 126°41′36′′E) is located in a parasitic scoria cone at an elevation of 508 m on the eastern side of Jeju Island, South Korea (Fig. 1). Continuous deposition on the crater floor has caused a hydrosere succession from lake to swamp during the last 4,000 years. Steep cone slopes hindered human access until the early 1900’s, and minimized disturbances to the crater. In 2006, the Mulyoungari swamp was declared a Ramsar site and has been well protected since under the Ramsar Convention and the Korean Wetland Conservation Act. Mulyoungari sediments are therefore quite appropriate for investigating MCA and LIA climates. A more detailed site description was provided in a previous pollen study^30^.

The Korean climate is characterized by four distinct seasons. The southeast summer monsoon gives the Korean peninsula hot and humid climate conditions while the northwest winter monsoon creates cold and dry conditions. In winter, a large high pressure cell develops over continental Siberia generating clockwise air circulation that flows over the peninsula. In summer, the wind direction is reversed as the surface air over the inland region becomes much warmer than the air above the ocean. Plenty of warm moisture is transported by southeasterly winds to the peninsula from the ocean. However, Jeju Island has a mild oceanic climate year round, with a smaller annual temperature range than the peninsula. The lowest and highest monthly mean temperatures (1980–2010) at the Seongsanpo station near the study site are 5.4 °C in January and 26.3 °C in August. The 1,967 mm mean annual rainfall at this station is the second highest among 74 stations in South Korea. Heavy rainfalls are concentrated during the summer monsoon season with ~55% of the annual precipitation amount recorded between July and September^32^ (Fig. S1).

During the summer, Jeju Island is considerably influenced by tropical cyclones known locally as typhoons^33^ (Fig. S1). These typhoons originate over the western Philippine Sea mostly between latitudes 10°N and 15°N. They begin to shift toward the southeastern coast of China. Some migrate onshore and dissipate. Others tend to turn from west to north, and then northeast around 30°N, with dominant southwesterlies. One or two typhoons travel across the southern part of the peninsula, and Jeju Island, between July and September. Typhoons usually have a stronger effect on Jeju Island since they often pass directly over the island. In addition, a couple of studies recently suggest that the number of typhoons affecting the peninsula tends to decline during El Niño (La Niña) events because of decreased (increased) SST of Western Tropical Pacific (WTP; 120–150°E)^34, 35^.

## Results and Discussion

### Mulyoungari diatom records

**Figure 2:**
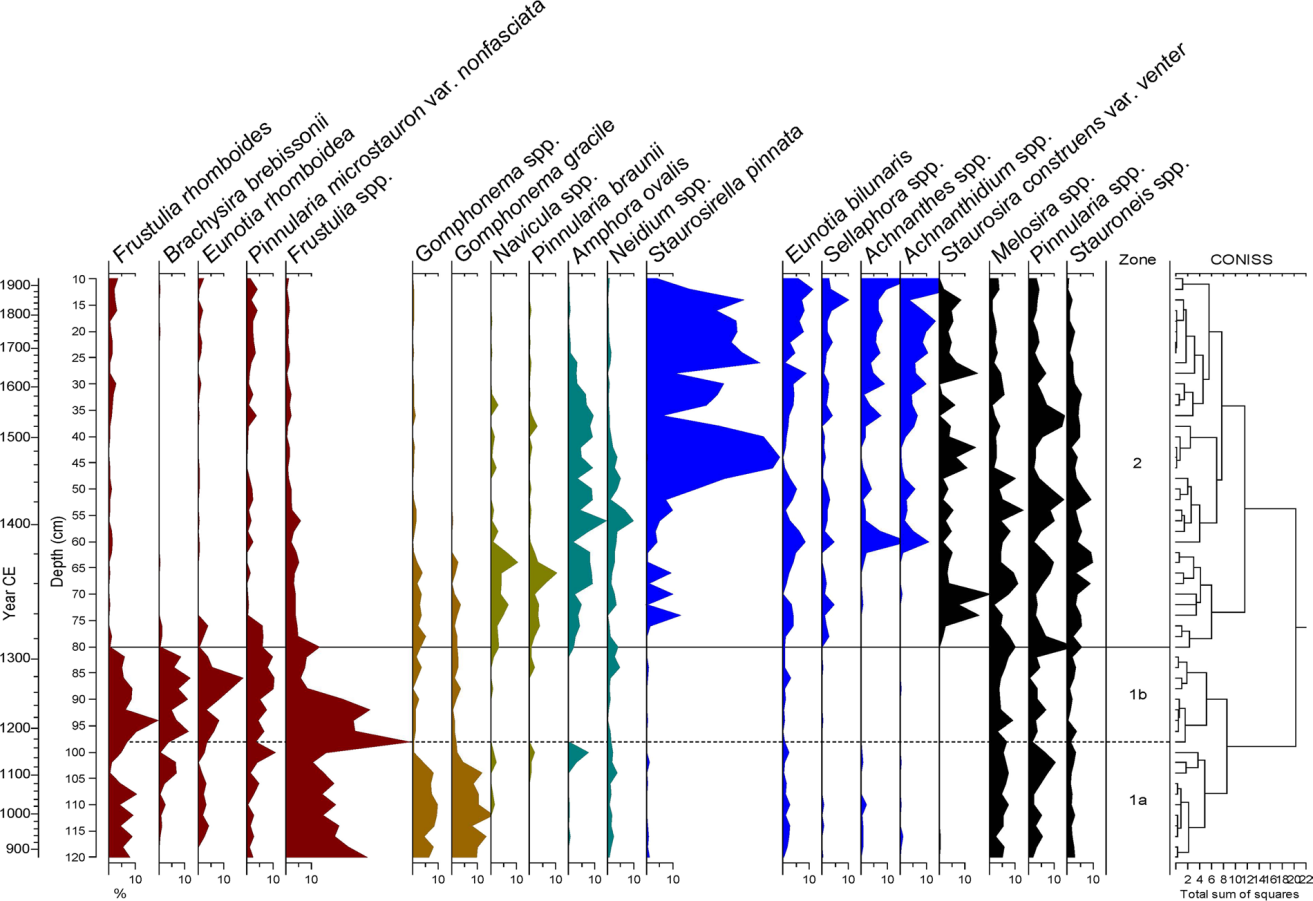
Selected diatom taxa from Mulyoungari sediments. Diatom taxa are assigned with different color shades according to their trophic preference. Diatoms in dark red, mocha, olive, teal, and blue likely prefer oligotrophic, less oligotrophic, mesotrophic, eutrophic, and more eutrophic conditions, respectively.

A stratigraphically constrained cluster analysis was carried out using CONISS, and three stratigraphic zones were delineated (Fig. 2). In zone 1a (900–1180 CE) and zone 1b (1180–1320 CE), *Frustulia* spp., which generally prefer oligotrophic acidic waters^36–38^ were particularly dominant. This may have been attributable to decreasing precipitation between 900–1320 CE. Dry climate likely reduced inward flux of nitrogen and phosphorus leading to oligotrophic conditions and a decline of algal productivity. Oligotrophication seems to have culminated in zone 1b given a marked increase in the frequency of *Frustulia* spp. Such change in lake trophic status is also similarly indicated by other oligotrophic diatom taxa including *Brachysira brebissonii*, *Eunotia rhomboidea*, and *Pinnularia microstauron* var. *nonfasciata* ^36, 37, 39^.

Zone 2 (1320–1920 CE) is characterized by the great abundance of *Staurosirella pinnata* after ~1400 CE. This taxa is commonly found in shallow eutrophic lakes^39, 40^. In the earlier part, there was a minor increase in *Navicula* spp. and *Pinnularia braunii*. *Gomphonema* spp. which lost its dominance in zone 1b also showed a slight rebound. *Amphora ovalis* and *Neidium* spp. thereafter became significant in the middle part. Benthic eutrophic taxa such as *Eunotia bilunaris* ^36^, *Sellaphora* spp.^41^, *Achnanthes spp*.^42^, and *Achnanthidium spp*. all began to rise from 1540 CE. Such changes over time in dominant taxa reflect that hydrosere succession to a swamp took place with steady eutrophication as terrigenous organic matter consistently flowed into the lake. A similar course of succession is also indicated by pollen data; for example, Cyperaceae percentages rapidly increased from 1540 CE^30^ (Fig. S2).

### Other Mulyoungari proxy records

**Figure 3:**
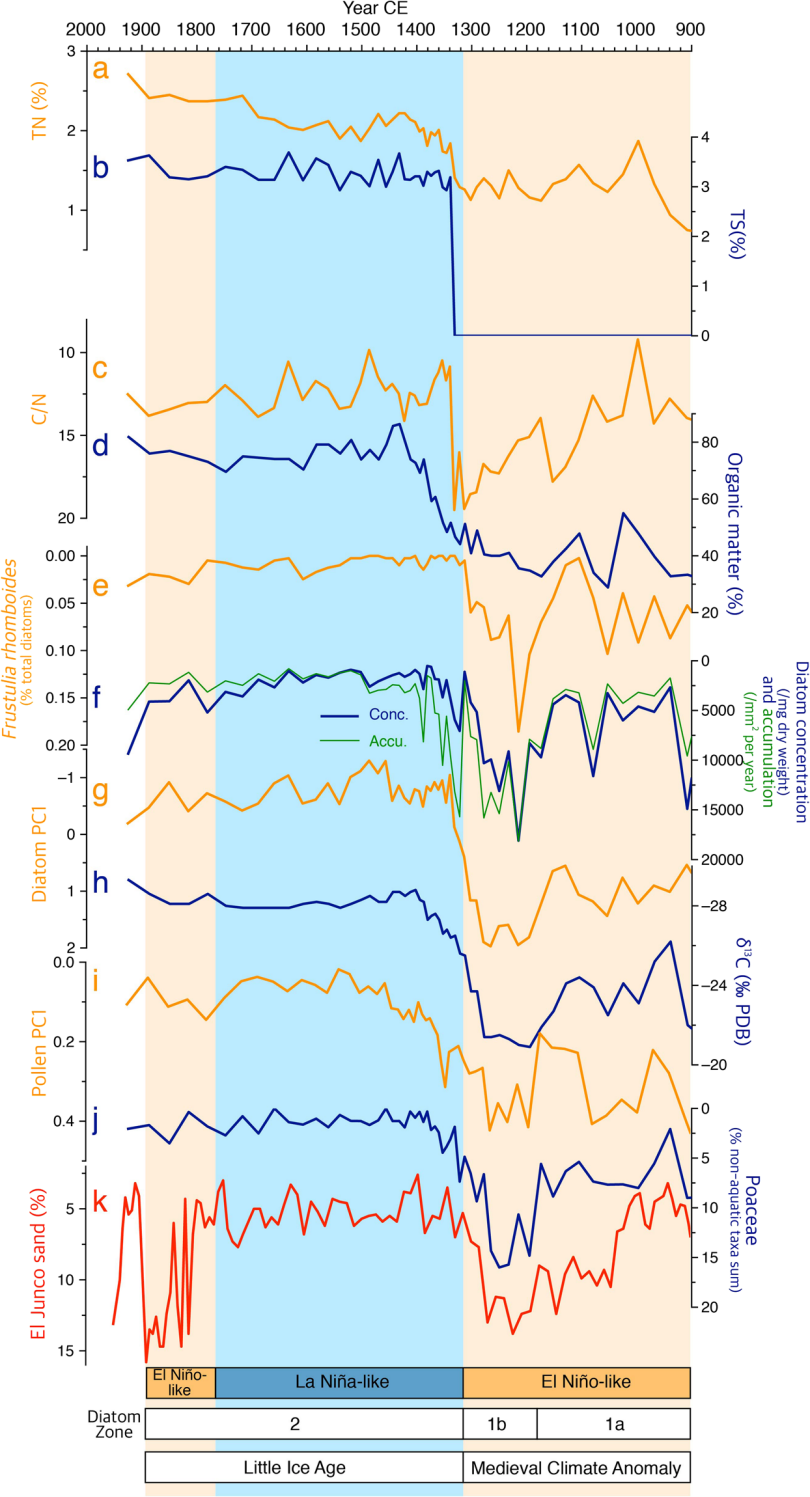
Comparison between Mulyoungari paleoenvironmental proxy data (**a–j**) and sand percentage records from El Junco Lake, Galapagos (**k**)^52^. (**a**) TN (%). (**b**) TS (%). (**c**) C/N ratios. (**d**) Organic matter (%). (**e**) *Frustulia rhomboides* (% total diatom). (**f**) Diatom concentration (blue) and accumulation (green). (**g**) Diatom PC1. (**h**) Stable carbon isotope ratios. (**i**) Pollen PC1. (**j**) Poaceae (% non-aquatic pollen and spore sum). (**k**) El Junko Sand %. Dry periods are indicated by orange boxes while wet periods by blue boxes.

In this study, many various proxy records are used to more accurately reconstruct the history of paleoclimate and paleolimnological change. Jeju climate variations during the MCA and LIA are well shown in the sedimentary records of diatom, total organic carbon (TOC), total nitrogen (TN), total sulfur (TS), and δ^13^C (Fig. 3). These records indicate that Jeju climate was relatively dry during the MCA (900–1320 CE). The driest conditions of the last millennium were likely present between 1180–1320 CE (diatom zone 1b) as implied in all the proxies. High C/N values in this period may have been associated with reduced algal abundance. C/N ratios are commonly used to determine the sources of sediment organic materials since the substantial difference is found between the C/N ratios of organic matter from vascular plants (>20) and from lacustrine algae (6–10)^43, 44^. A rise of C/N values up to 20 does not seem to have been caused by the expansion of terrestrial biomass given a simultaneous decrease in sedimentary organic content (Fig. 3d). This therefore indicates a decline in algal production arising from lowered precipitation and enhanced oligotrophic conditions.

Like C/N ratios, δ^13^C has been also used to estimate the relative importance of each organic matter source in lake sediments. Land plants that use the C4 and C3 pathway of photosynthesis generally produce organic matter with δ^13^C values ranging from −14 to −10‰ and −30 to −22‰, respectively^45, 46^. Lake algae much more strongly discriminate against^13^C so that they have relatively depleted δ^13^C values, usually between –31 and –26‰^43^. Therefore, δ^13^C increases to −20‰ in zone 1b reflect that C4 plants had advantages over C3 plants around the study site as climate became drier between 1180–1320 CE. The possibility of expanding C4 plants during this period is also supported by a pronounced increase in Poaceae percentages (Fig. 3j).

**Figure 4:**
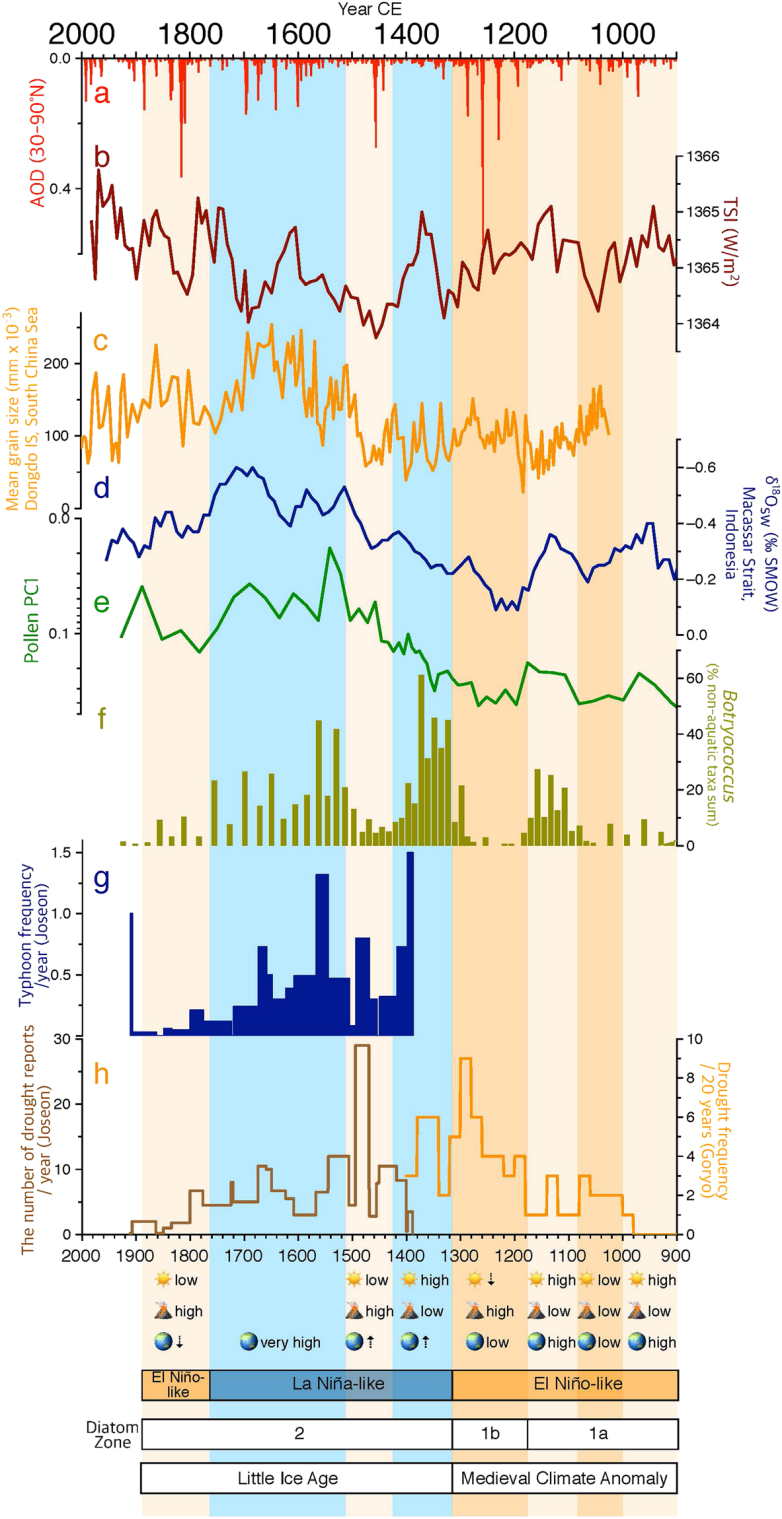
Comparison among 30–90°N aerosol optical depth (AOD)^58^ (**a**), total solar irradiance (TSI)^57^ (**b**), mean grain size of sediments from Dongdao Island, South China Sea^59^ (**c**), δ^18^O_sw_ of marine sediment cores (BJ8) from the Makassar Strait, Indonesia^60^ (**d**), pollen PC1 (**e**) and *Botryococcus* percentage records^30^ (**f**) from Mulyoungari sediments, and historical typhoon frequencies^64^ (**g**) and drought frequencies^63, 64^ (**h**) during the past millennium in the Korean peninsula. Different time units are used in the diagram of drought frequencies (Fig. 4h) because the data are obtained from two different historical documents: Goryosa (918–1392 CE) and Annals of the Joseon Dynasty (1392–1863 CE). Note the log scale in pollen PC1. Dry (wet) periods are demonstrated by orange (blue) boxes. Beige boxes are indicative of less dry conditions. The simplified conditions (high, low, increasing ⇡, or decreasing ⇣) of solar activity , volcanic activity, and WTP SSTs are shown for each time period at the bottom. This diagram is produced using pro Fit 7.0.7 software (www.quansoft.com).

The results of principle component analysis of diatom assemblages also show the hydroclimate change during the last millennium. The first principle component (PC1) can be considered as an indicator of lake trophic status given its positive relationship with highly oligotrophic, acidophilous taxa (*Frustulia rhomboides*)^36^. High values of PC1 in zone 1b therefore again indicate prevailing dryness and lake oligotrophication between 1180–1320 CE. However, under drier conditions, diatoms seem to have been more competitive than other algae such as *Botryococcus* (Fig. 4f). Their concentration and accumulation rates both culminated in zone 1b, reflecting that diatoms were generally favored by a dry climate.

However, in zone 2, diatoms lost their dominance as increasing precipitation led to enhanced influx of terrigenous organic matter. Such competitive weakness of diatoms would have been associated with declining Si/P ratios in the lake water^47^. In contrast, there was a conspicuous increase in *Botryococcus* from 1320 CE, as indicated by the previous pollen study^30^ (Fig. S2). Anoxic conditions at the lake bottom arising from the vigorous decomposition of organic matter^48^ are implied by a sudden rise of TS at 1340 CE.

Throughout the entire period of the investigation, there are robust correlations between PC1 records of pollen and diatoms, Poaceae percentages, and δ^13^C values (Fig. 3). Pollen PC1 values seem to indicate rainfall variability like δ^13^C values given its consistent relationship with desiccation tolerant Poaceae. The link between pollen and diatom records clearly implies simultaneous responses of lake environment and surrounding forests to climate variations during the last millennium.

### The dry MCA/wet LIA pattern

A detailed examination of previous studies from East Asia is necessary to understand the mechanisms underlying the dry MCA and wet LIA patterns in the study area. It is generally agreed that over monsoonal East Asia relatively warm conditions dominated during the MCA while cool conditions did so during the LIA^24, 49^. This is principally attributable to climate response to late Holocene precession, sunspot activity, and volcanic eruptions. It has often been suggested that more southerly positions of the ITCZ during the LIA brought less precipitation to northern China and more to southern China, and vice versa during the MCA^25, 26^. However, since our results are similar to proxy records from southern China (despite a higher latitudinal position of the study site), we need to consider the possibility that another factor significantly modulates Jeju climate. Recent studies have already suggested that Jeju Island’s mid-to late-Holocene climate was strongly linked to SST variations in the tropical Pacific^30, 31, 50^. The climate in coastal East Asia seems to have been more substantially driven by WTP SST variability than by orbital precession or solar forcing, unlike other inland areas in East Asia^49, 51^.

The possible link of Jeju climate with tropical Pacific SSTs is well supported by robust relationships between our Mulyoungari multiproxies and lake sedimentary records from the Galapagos islands^52^ (Fig. 3). Sand percentage data from El Junko lake demonstrate a dry LIA and wet MCA in the eastern tropical Pacific (ETP) with particularly high precipitation between 1180–1320 CE, reflecting a significant relationship between ETP SSTs and Jeju climate. Given current ENSO phenomena in the ETP, the MCA would have been predominated by El Niño-like conditions while the LIA by La Niña-like conditions. However, contrasting results between studies using observed and proxy records make it difficult to determine the mechanism underlying the linkage between tropical Pacific SSTs and the climate in coastal East Asia. For example, East Asia has been suggested to have warmer winters during El Niño events as SST cooling in the western North Pacific (WNP) diminishes the pressure gradient between the continent and ocean^28^. Persistent anticyclones in the following summers also likely strengthen summer monsoons leading to positive rainfall anomalies^53^. However, recent paleolimnological studies indicated that during El Niño-like conditions, a decrease in WTP SSTs weakened cyclones over the WNP and consequently reduced moisture transfers to East Asia^31, 54–56^. Such paleoclimate records therefore suggest that the influence of long term ENSO-like variability on East Asia cannot be fully explored based on interannual-scale ENSO and East Asian summer monsoons (EASM) shown in weather observations and model simulations.

### Hydroclimate variability of the last millennium and typhoons

It is believed that Holocene climate in the Northern Hemisphere was mainly controlled by orbital precession, sunspot activity, and volcanic eruptions. Our results also indicate that the climate of the past millennium in the study area was significantly modulated by solar radiative forcing^57^ and volcanic activity (reflected in reconstructions of aerosol optical depth)^58^. *Botryococcus* percentages^30^ and PC1 of pollen data are indicative of the relatively dry conditions between 1000–1080 CE, 1180–1320 CE, 1420–1510 CE, and 1760–1880 CE. These dry periods all occurred along with increasing solar output and reduced volcanic activity except between 1000–1080 CE (Fig. 4).

The PC1 of Mulyounagri pollen records implies again that Jeju climate was greatly influenced by tropical oceanic forcing. Strong linkages are observed between the PC1 data and grain size sedimentary records from Dongdao island, South China Sea^59^ and marine δ^18^O data off Borneo island^60^. It reflects that similar climate changes were induced in these distant areas by the Kuroshio current and monsoon circulation. As mentioned earlier, ENSO like variations likely caused a dry MCA and wet LIA in the WNP and coastal East Asia. Interestingly, there was a long and persistent duration of wetness between 1520–1760 CE despite being interfered with by the Maunder minimum (1640–1720 CE), which was the coolest time period over the last millennium in the Northern Hemisphere. The “Annals of Joseon Dynasty”^61^, the annual historical records from 1392 to 1863 CE, indicate that increased extreme climate events during 1670–1671 and 1695–1696 led to the great Gyeongsin famine and Eulbyeong famine, respectively. However, according to the “Annals of Joseon Dynasty”, droughts do not seem to have played a main role in bringing such severe famines. It is more likely that these famines were caused by an increased number of typhoons and consequently more frequent floods. This historical text clearly states that tremendous damages to life and property across the peninsula resulted from extraordinary number of consecutive storms and floods between June and October of 1670 CE (see Fig. 4h). The “Munhunbigo”, another historical text of the Joseon Dynasty, also shows that the Korean peninsula received relatively high precipitation between 1550–1900 CE^62^.

Historical data for the last millennium (“Goryosa” and “Annals of Joseon Dynasty”)^63, 64^, pollen and algae data uniformly indicate droughts between 1180–1320 CE, a decreased number of typhoons and consequent dryness between 1420–1520 CE, and wet conditions between 1520–1760 CE (Fig. 4). The link between them reflects that the same climate variations were experienced by Jeju island and the peninsula. However, the northern part of the peninsula was probably less influenced by oceanic forcing than the southern part. This is clearly indicated by the great difference between pollen records from Maar Lakes close to the northern border of North Korea^65, 66^ and from Jeju Island^30, 31^. Climate variability would have been spatially different in the peninsula due to possible latitudinal discrepancy in oceanic influence.

### The role of typhoons as an agent for climate teleconnections

**Figure 5:**
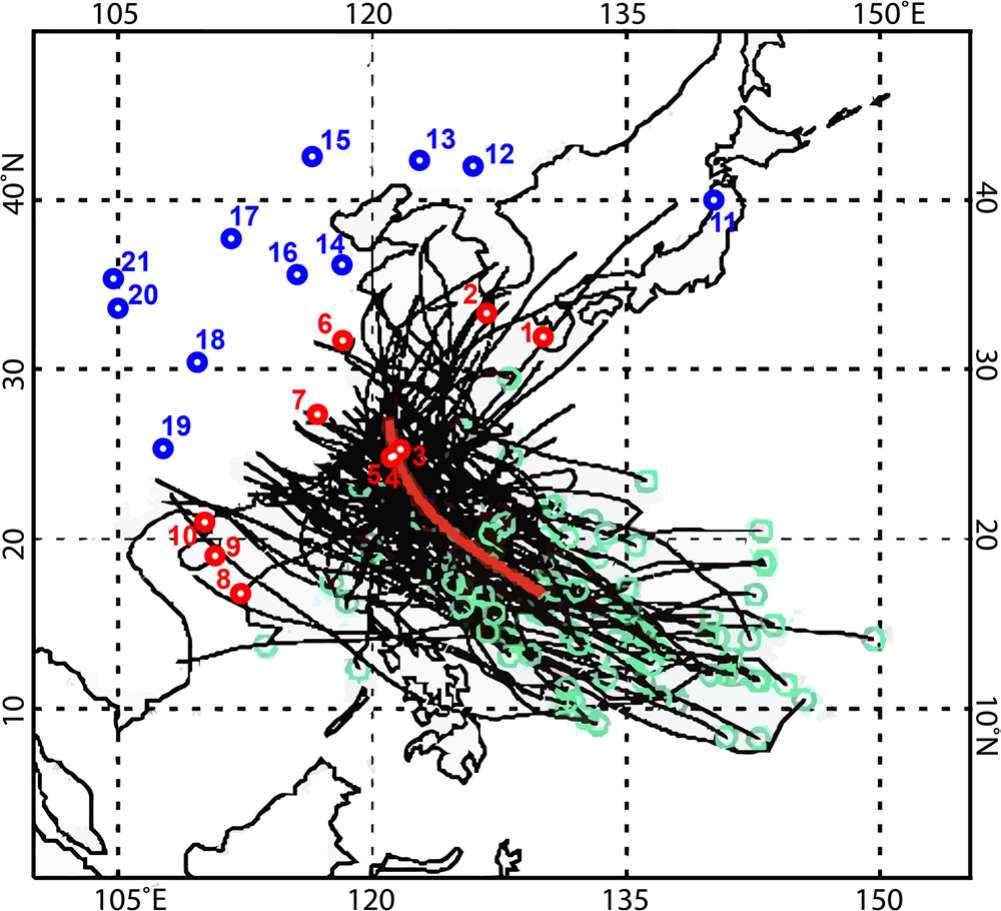
Locations of paleohydroclimate records examined in this study. Coastal sites showing the dry MCA/wet LIA pattern are indicated by red numbers (1–10) while other sites are by blue numbers (11–21). Dr. Seo, K.H. provided the background map with tropical cyclone tracks, which was created using an Interactive Data Language program (IDL 8.1; www.harrisgeospatial.com). These tracks belong to the second cluster according to his classification of tropical cyclones between 1979 and 2013^69^. The black lines and red thick line indicate the individual tropical cyclone tracks and the vector weight, respectively. The green open circles show the tropical cyclone genesis positions. The frequency of tropical cyclones in this cluster is significantly negatively correlated with ETP (the NINO3 region) SSTs during El Niño conditions^69^.

A dry MCA and wet LIA have been suggested by previous coastal studies in East Asia; for example, Taiwan^67, 68^, east coast of China^12, 16^, southwest Japan^22^. These study sites are all located near to the route followed by typhoons affecting Jeju. Kim and Seo’s recent study on tropical cyclone tracks in the WNP^69^ demonstrates a strong negative correlation (r = −0.37) between ETP SSTs and the frequency in tropical cyclones mostly starting from western Philippine Sea (Lat. 10N–15N; Long. 125E–140E)^69^. These tropical cyclones tend to successively hit northern Philippines, Taiwan, the east coast of China, and Jeju island (Fig. 5). Although another tropical cyclone cluster in their classification is more important to Jeju island and the peninsula, it has no significant relationship with eastern or central tropical Pacific SSTs. According to their study, positive anomalies of ETP SSTs (El Niño like conditions) lead to ~40% decrease in the frequency of tropical cyclones in the aforementioned tracks^69^. This reflects that less tropical cyclones were generated in the WNP because of El Niño-induced cooling SSTs and thereby high pressure anomalies over the western Philippine Sea. Precipitation at Jeju island is modulated by the number of typhoons passing nearby and, more importantly, by the amount of moisture transferred via westerlies during the extratropical transition of typhoons in China^70^. Therefore, a considerable rainfall decrease could be caused by declining typhoon generation over the western Philippine Sea attributable to El Niño patterns.

Tropical oceanic forcing needs to be more carefully explored to understand the driving forces behind a dry MCA and wet LIA in coastal East Asia and in the WNP. As mentioned earlier, it is generally agreed that the varying EASM strength caused differences in hydroclimate patterns between northern and southern China. However, typhoons also seem to have exerted a significant influence on East Asian climate establishing an interesting pattern of dry MCA and wet LIA in coastal East Asia (Fig. 5, Table 1). Therefore, during the investigation of the past and future climate change in East Asia, coastal areas should be considered as a separate hydroclimate region that shows a stronger link to tropical Pacific SSTs and typhoons.

**Table 1:** Paleohydroclimate records used in this study.

Site number	Site name	Latitude (°N)	Longitude (°E)	Proxies	References
**1**	Lake Kaiike	30.85	129.87	Sediment δD	Soelen *et al*.^22^
**2**	Mulyoungari Swamp	33.37	126.68	Pollen, diatom, δ^13^C, CN, TS	This study
**3**	Southern Okinawa Trough	24.80	122.49	Diatom	Li *et al*.^75^
**4**	Dahu Lake	24.75	121.70	Pollen, diatom, grain size	Wang *et al*.^68^
**5**	Tsuifong Lake	24.50	121.60	Diatom, δ^15^N, δ^13^C, magnetic susceptivility	Wang *et al*.^67^
**6**	Lower Huai River and Yangtz River Basin	32.36	117.84	Historical documents	Man (2009)^16^ (as cited in ref. 25)
**7**	Jiang-Nan Area	27.50	117.00	Historical documents	Zheng *et al*.^12^
**8**	Dongdao Island	16.75	112.80	Grain size	Yan *et al*.^59^
**9**	Hainan Island	19.30	110.67	Coral δ^13^C and δ^18^O	Deng *et al*.^76^
**10**	Huguangyan Maar Lake	21.15	110.28	TOC, TN, Sr	Zeng *et al*.^19^
*11*	Lake San-No-Megata	39.93	139.70	Geochemical data, magnetic susceptibility	Yamada *et al*.^18^
*12*	Xiaolongwan Lake	42.30	126.35	Sediment δ^13^C	Chu *et al*.^15^
*13*	Maili Pond	42.87	122.88	Pollen	Ren (1998)^11^
*14*	Kaiyuan Cave	36.40	118.03	Stalagmite δ^18^O	Wang *et al*.^77^
*15*	Dali Lake	43.26	116.60	TOC	Xiao *et al*.^54^
*16*	North China	36.40	115.12	Historical documents	Man (2009)^16^ (as cited in ref. 25)
*17*	Gonghai Lake	38.90	112.23	Geochemical data	Liu *et al*.^20^
*18*	Heshang Cave	30.45	110.42	Stalagmite δ^18^O	Hu *et al*.^51^
*19*	Dongge Cave	25.28	108.08	Stalagmite δ^18^O	Zhao *et al*.^21^
*20*	Wanxiang Cave	33.58	105.00	Stalagmite δ^18^O	Zhang *et al*.^14^
*21*	Longxi Area	35.45	104.78	Historical documents	Tan *et al*.^13^

Coastal sites showing the dry MCA/wet LIA pattern are indicated by bold numbers (1–10) while other sites are by italic numbers (11–21) (see Fig. 5 for the location of each site).

### Droughts from 1180 to 1320 CE

Mulyoungari multiproxy records indicate that the most severe droughts over the last millennium occurred between 1180–1320 CE. This dryness, which was possibly attributed to an intense volcanic activity (Fig. 4a) and consequent Pacific SST changes, would have led to widespread famines in the country. Moreover, the political environment during this period was consistently unstable since military officers ruled the kingdom of Goryo for 100 years after dethroning the reigning king at 1170 CE and the Mongol empire launched consecutive assaults between 1231–1258 CE. Such political instability presumably left people extremely vulnerable to the impact of climate deterioration.

Conversely, a recent tree ring study indicates that warm season precipitation increased in central Mongolia during the same period^5^. The Mongol conquest of Eurasia would have been substantially favored by temporary climate amelioration in the steppe region during the13^th^ century because productive grasslands would likely supply sufficient food to feed war horses^5^. It may have been a tragedy for destitute Goryo people to defend against strong Mongol armies that benefited from the contrasting climate conditions between central Mongolia and coastal East Asia. The kingdom of Goryo was a vassal state of the Mongol Yuan Dynasty for about 80 years after finally losing the Goryo-Mongol war in 1259 CE.

### Summary and implications

Our results indicate that the climate in coastal East Asia was principally controlled by ETP SST change and ENSO like variations during the past millennium. Since the MCA was dominated by persistent El Niño like patterns (and the LIA by La Niña patterns)^52, 71, 72^, coastal East Asia would have experienced decreasing precipitation during the MCA and increasing precipitation during the LIA. There are also quite a few previous coastal records that show similar hydroclimate patterns (Table 1). However, recent reviews^25, 26^ do not seriously evaluate the role of tropical Pacific forcing for the East Asian climate over the last millennium. More attention needs to be paid to the variation in ETP (the NINO3 region) SSTs for a better understanding of past climate change in coastal East Asia. Our results also suggest that SST anomalies in the tropical Pacific should be closely assessed to reduce uncertainty in predicting hydroclimate change in coastal East Asia.

## Materials and Methods

### Core materials and chronology

**Figure 6:**
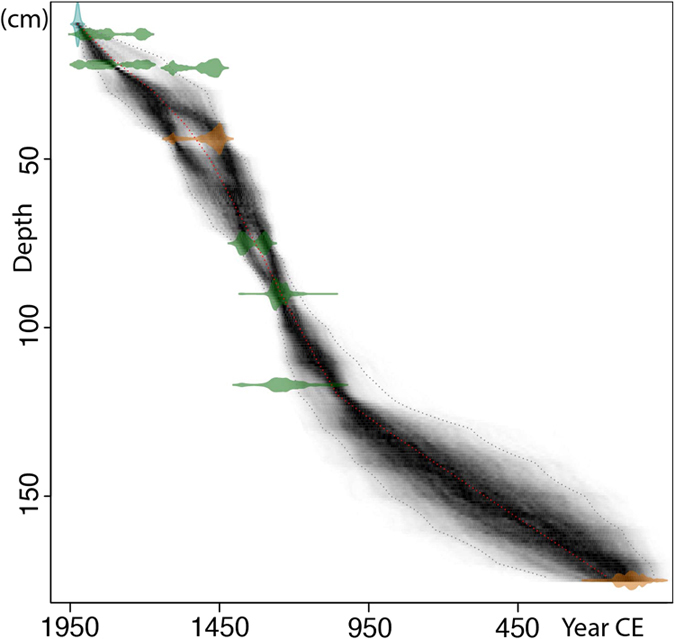
The Mulyoungari sediment core age depth profile. The previously reported radiocarbon dates were used for the age depth model^30^. Age probability distributions are plotted in light brown for bulk sediment samples and in green for plant samples. The best age depth model (red dot line) with a 95% confidence interval (gray dot line) was established based on Bayesian principles using Bacon 2.2^74^.

In 2014 we recovered a 4 m long sediment core from the Mulyoungari swamp with a Russian type peat corer (Fig. 1). Previous study of the core established its whole chronology using 14 radiocarbon dates^30^. In the present study, sediments between the depths of 10 and 120 cm were selected for detailed multiproxy analyses to determine the hydroclimate variations of the last millennium. The chronology for this study was based on a total of 8 radiocarbon dates and an estimated age for the top (25 cal yr BP)^30^ (Fig. 6).

### Multiproxy and historical data

A total of 56 samples were taken for diatom analysis at 2 cm intervals. The samples were treated with hydrochloric acid (5%) and hydrogen peroxide (10%) to remove carbonates and organic materials, respectively. Sediment disaggregation was performed using calgon solution (sodium hexametaphosphate). Diatom counts were made on a Leica microscope (DM2500) with a 100x objective at total magnification of 1000x. A minimum of 400 diatom valves were counted from each slide.

Total organic carbon, nitrogen, and sulfur contents were measured on HCl treated samples at 2 cm intervals using a Flash EA 1112 element analyzer. Samples for stable carbon isotope analysis were also taken from the same depths and treated with 5% HCl to remove any carbonates. Carbon isotope ratios in the sediment organics were determined using a GV Isoprime mass spectrometer at the Korea Basic Science Institute. Replicate analyses of the samples gave a precision of <± 0.2‰. Pollen and algae data that were previously reported^30^ were also used for this study.

Principal component analysis (PCA) was applied to the pollen and diatom percentage data to extract the main trends using C2 1.4 software^73^. We used taxa with a relative abundance >2% in at least two samples for the analysis. The percentages were square root transformed in an attempt to stabilize the variance. Historical documents were also used for this study. Drought records for the Goryo Dynasty (918–1392 CE) were obtained from the “Goryosa”^63^ while drought and typhoon records for the Joseon Dynasty (1392–1910 CE) were from the “Annals of the Joseon Dynasty”^64^.

## Electronic supplementary material

supporting information
