# Supplementary material for: The Link between ENSO-like Forcing and Hydroclimate Variability of Coastal East Asia during the Last Millennium
Source: Sci Rep. 2017 Aug 15;7:8166. doi: 10.1038/s41598-017-08538-1 (PMC5558021; doi:10.1038/s41598-017-08538-1)
Supplement: Supplementary file 1 — supporting information [file 41598_2017_8538_MOESM1_ESM.pdf]

Supplementary Information

The Link between ENSO-like Forcing and Hydroclimate Variability of Coastal East Asia during the Last Millennium

Jungjae Park [a, b, *], Jiwoo Han [a], Qiuhong Jin [a], Junbeom Bahk [a], Sangheon Yi [c]

[a] Department of Geography, Seoul National University, Sillim-dong, Gwanak-gu, Seoul, 151-742, Republic of Korea

[b] Institute for Korean Regional Studies, Seoul National University, Sillim-dong, Gwanak-gu, Seoul, 151-742, Republic of Korea

[c] Geo-Environmental Hazards & Quaternary Geology Research Center, Korea Institute of Geoscience and Mineral Resources, Daejeon, 305-350, Republic of Korea

* Corresponding author

Email address: jungjaep@snu.ac.kr

Telephone number: 82-62-530-2687

Fax number: 82-62-530-2689

SI Figures

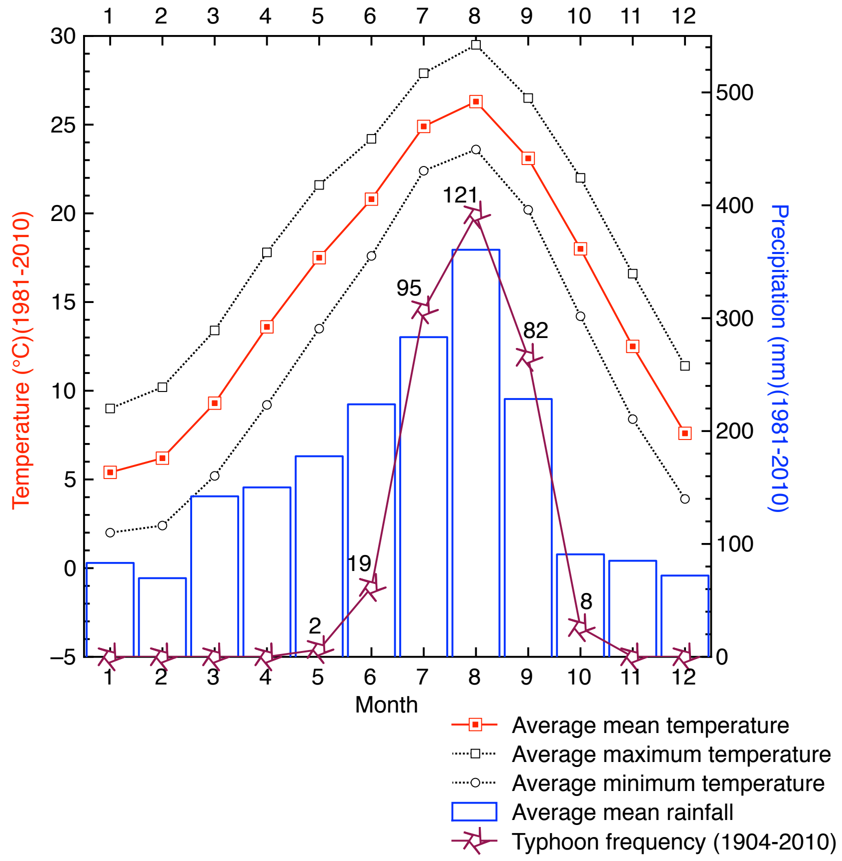

Fig. S1. Climate data for Seongsanpo, Jeju Island[1]. The numbers within the diagram indicate monthly frequency of total typhoons affecting Korean peninsula between 1904-2010[2]. This diagram is produced using pro Fit 7.0.7 software (www.quansoft.com).

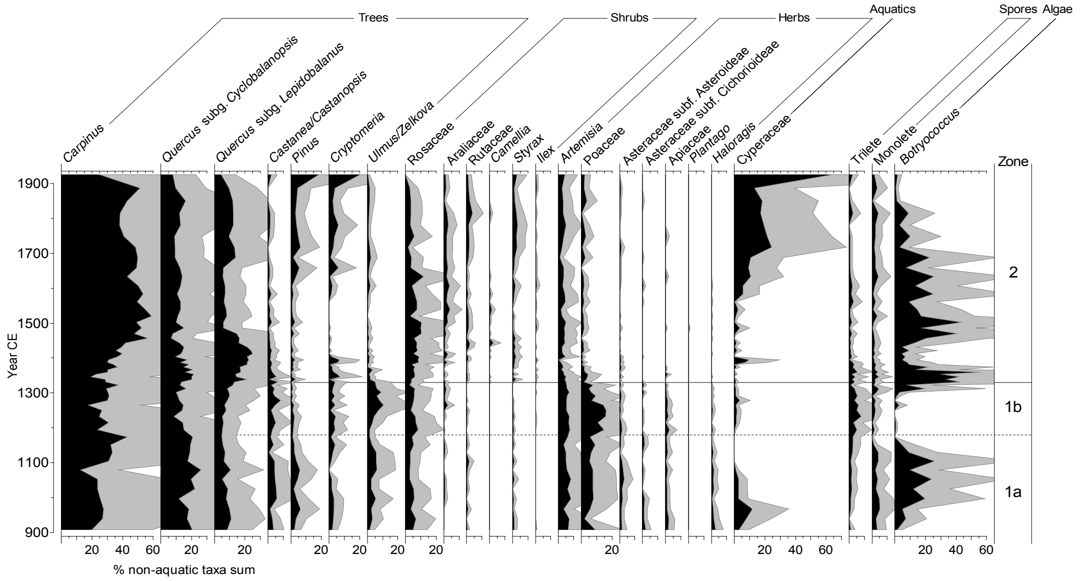

Fig. S2. Selected pollen taxa from Mulyoungari sediments[3]. All percentages are based on the total non-aquatic taxa sum. This diagram is produced using Tilia 2.0.41 software (www.tiliait.com).

References

1 *Domestic Climate Data. Korea Meteorological Administration*, <http://www.kma.go.kr/weather/climate/average_30years.jsp> (2015).

2 Anonymous. *Typhoon White Book.* (Korea Meteorological Administration, 2011).

3 Park, J., Shin, Y. H. & Byrne, R. Late-Holocene vegetation and climate change in Jeju Island, Korea and its implications for ENSO influences. *Quaternary Science Reviews* **153**, 40-50 (2016).
